# Current data science capacity building initiatives for health researchers in LMICs: global & regional efforts

**DOI:** 10.3389/fpubh.2024.1418382

**Published:** 2024-11-27

**Authors:** Agklinta Kiosia, Sally Boylan, Matthew Retford, Larissa Pruner Marques, Flávia Thedim Costa Bueno, Christine Kirima, Md Saimul Islam, Aliya Naheed, Anne Wozencraft

**Affiliations:** ^1^Health Data Research UK (HDR UK), HDR Global, London, United Kingdom; ^2^Division of Diabetes, Endocrinology and Metabolism, Imperial College London, London, United Kingdom; ^3^Oswaldo Cruz Foundation (Fiocruz), Rio de Janeiro, Brazil; ^4^The Global Health Network, University of Oxford, Oxford, United Kingdom; ^5^Non-Communicable Diseases, Nutrition Research Division, icddr,b, Dhaka, Bangladesh

**Keywords:** data science, capacity building, global health research, low-and middle-income countries, health researchers

## Abstract

**Background:**

Data science approaches have proved crucial for generating major insights to address public health challenges. While such approaches have played significant roles during the COVID-19 pandemic, there has been limited investment in capacity building in data science skills and infrastructure for health researchers in LMICs.

**Objectives:**

This review aims to identify current health data science capacity building initiatives and gaps in Africa, Asia, and Latin America and the Caribbean (LAC), to support knowledge sharing and collaborations, and inform future initiatives and associated investment.

**Methods:**

We conducted a literature review using PubMed and Scopus, supplemented by a grey literature search on Google to identify relevant initiatives. Articles were screened based on inclusion criteria.

**Findings:**

From 212 records, 85 met inclusion criteria, with 20 from PubMed and Scopus, and 65 from grey literature. The majority of programmes are tailored to specific disease areas, varying by region. Despite these efforts, there are limited initiatives with a clear, documented strategy on data science capacity building to accelerate global research insights, with the majority adopting a fragmented approach.

**Conclusion and future directions:**

Despite the integration of data science approaches into health research initiatives in LMICs, there is a need for a standardised framework on data science capacity building to facilitate multidisciplinary and global collaboration. Structured approaches, inter-disciplinary, inter-regional connections and robust impact measurement will all be vital for advancing health research insights in these settings.

## 1 Introduction

Over the past two decades, rapid technological advances have reshaped the role of data science in health research, bringing innovative insights and solutions to tackle critical health and societal challenges, particularly in low-and middle-income countries (LMICs). These have included the development of disease surveillance and early warning systems for infectious diseases, precision health interventions tailored to local contexts, and optimised resource allocation strategies in public health through health data analytics (1–4). LMICs face unique healthcare and societal challenges, often characterised by limited work force, inadequate health budget, fragile health systems, and complex socioeconomic factors that contribute to persisting disease burdens and inequalities (5, 6). Within this complex landscape, data science emerges as a transformative tool to bridge gaps and drive positive change in public health.

The term “data science” refers to a multidisciplinary field that incorporates a range of methodologies, techniques, algorithms and processes to analyse and draw conclusions from data (7). In the context of health, data scientists use these tools to generate health insights, advance our understanding of health and disease, and ultimately improve health outcomes. Data science methods used in health research can provide actionable insights derived from diverse data sources that enable targeted interventions (8–10), efficient resource allocation (11, 12), and evidence-based decisions to improve the management of diseases and health outcomes of populations and communities (3, 13).

To fully leverage the potential of data science capabilities for health research in LMICs, it is imperative to take a holistic approach to building data science capacity. Capacity building is defined by the United Nations as “*the process of enhancing the skills, resources, and adaptive capabilities of organisations and communities, enabling them to not only cope with change, but also to thrive in a rapidly evolving environment in a sustainable manner*” (14). This approach extends beyond individual skills development and necessitates the establishment of robust institutional frameworks and supportive policies. It has increasingly been recognised that data science capacity building is integral to sustainable development in LMICs, with growing investment in education, training and infrastructure to enhance capabilities for individuals, communities and institutions (15–22). As part of this wider data science capacity building ecosystem, mentorship and sustainable leadership and capability strengthening efforts are key in fostering a supportive environment, empowering new research leaders to contribute to data-driven decision-making (15, 23, 24). Furthermore, establishing data governance mechanisms, robust institutional frameworks, data sharing and data access policies can ultimately enhance data quality, privacy and interoperability, and encourage a culture of data collaboration between research teams, institutions and countries (24, 25). A vital element in driving effective data science capacity building for health research is the development of supportive policies that prioritise investment in data infrastructure, regulatory frameworks that promote ethical data use, and open data principles that ensure trustworthy and responsible data practices (25).

Despite the increased global commitment to data science capacity building efforts in LMICs, the practical implementation of this endeavour poses significant challenges. This is evidenced by the scarcity of scholarly publications, insufficient training and graduate programmes dedicated to examining and addressing data science capacity building for health research in LMICs, which is influenced by a multitude of factors (20). Challenges to effective capacity building span the domains of politics, socioeconomics and technology, and include limited access to education, lack of specialised technical infrastructure and sustainable financial mechanisms (26), workforce attrition due to the brain drain (up to 70%) (15, 26), and the persistent lack of translating research evidence into policies and practices (27). The lack of resources, mentorship, specialised training and continuous professional development in health data science research, and its integration into policy making, highlight the urgent need for targeted interventions that address these barriers and foster a sustainable ecosystem for data science capacity building in LMICs (19).

The primary objective of this review is to identify and present an overview of the current landscape of regional and global data science capacity building initiatives that are relevant and accessible to global health researchers across Africa, Asia and Latin America & the Caribbean (LAC). The selection of these regions was guided by a partnership initiative that involved global partners in Africa, LAC, and South Asia.

The second objective is to identify gaps and the key success factors that ensure sustainability and impact of these initiatives, and the third objective is to make recommendations to improve coordination and serve as a catalyst for action and further exploration by stakeholders in the field, including individual researchers, funders and organisations planning to develop and launch related initiatives Overall, this should encourage collaboration, coordination and investment in related capacity-building efforts that may contribute to the development of a common global framework for health data science skills.

## 2 Methodology

### 2.1 Definitions of health data science capacity building initiatives and programmes

For this review, health data science capacity building initiatives and programmes have been defined as those affiliated to organisations, institutes or networks/consortia that focus on accelerating health research activities in LMICs for public benefit. Notably, this definition extends beyond a narrow focus on formal university courses, focusing on dynamic initiatives and programmes that transcend traditional academic structures, placing particular emphasis on open access resources that are available to individual researchers and organisations, and contribute to strengthening of the global health data science ecosystem. These initiatives aim to foster a practical and applicable understanding of health data science approaches, emphasising real-world impact and engagement with diverse stakeholders. By broadening the scope beyond formal education settings, the review aimed to capture a comprehensive range of initiatives and programmes that contribute significantly to the advancement of health data science research capabilities in LMICs.

### 2.2 Search strategy for reviewing the literature

To identify ongoing health data science capacity-building programmes relevant to health researchers in LMICs, with either a global or regional focus on Africa, Asia and LAC, the initial intent was to conduct a formal systematic or scoping review. However, due to the limited published literature on this topic - as it is not commonly explored in academic research - the approach was adjusted to follow an exploratory search strategy. Between 20th July 2023 and 30th January 2024, an extensive literature review was conducted using PubMed and Scopus as primary databases. The main search strategy involved meticulous filtering based on geographical location, ensuring the coverage of targeted LMICs and regions, and filtering publications between January 2019 and December 2023. The review period was selected to capture a wide range of relevant studies and reports from the past 5 years. This timeframe allowed us to include data both before and after the COVID-19 pandemic, providing insights into how the pandemic has influenced health data science capacity building. Additionally, many organisational strategy reports typically cover a 3 to 5-year span, ensuring that our review encompassed the latest strategic developments and their impact, when available. This process involved setting the timeline of publications between the afore mentioned timeframe and combining selected keywords and terms, such as capacity building/strengthening, global health, health data, health data science, health data research, initiatives, consortium, network, LMICs, Africa, LAC, South-East Asia, South Asia, low-resource settings, digital health, training, data science hub, healthcare, disease surveillance, data management, genomics, bioinformatics, infectious disease, non-communicable disease, nutrition, maternal health, childhood health, and health systems. The string search information can be found in Supplementary Table S1.

To complement this research strategy, the inquiry was extended to grey literature through Google searches, utilising various combinations of the aforementioned keywords and terms. Additionally, a snowballing sampling technique was adopted to uncover further health data science capacity-building initiatives. This involved identifying initiatives mentioned on the websites of known organisations and cross-referencing these findings with citation searches of articles mentioned on identified websites, or initiatives discovered through references in research papers. To minimise selection bias, the findings were reviewed by a diverse group of authors based in Africa, Asia, LAC, and Europe, all affiliated to the partnership initiative.

### 2.3 Inclusion and exclusion criteria

Inclusion:

1. Active programmes/initiatives during the search period previously specified (January 2019–December 2023), operating in LMICs, with a specific emphasis on Africa, South East Asia, South Asia and LAC
2. Global health-focused programmes or initiatives
3. Readily accessible for individual researchers and health practitioners
4. Programmes/initiatives supported by reputable organisations/institutes and funders with strong presence in LMICs
5. Publications composed in the English language
6. Programmes/initiatives dedicated to enhancing data science skills
7. Inclusion of a broad spectrum of health science domains and programmes (e.g., disease/pandemic surveillance, nutrition, infectious and non-communicable disease, genomics, healthcare systems).

Exclusion:

1. Programmes/initiatives that have been discontinued during the literature search (January 2019–December 2023)
2. Organisations with outdated websites or inactive status during the literature search
3. Materials in languages other than English
4. Programmes/initiatives with a primary focus outside LMICs
5. Programmes/initiatives centred on areas other than health research
6. Initiatives exclusively dedicated to academic programmes, such as diplomas and degrees.

## 3 Findings

**Figure 1:**
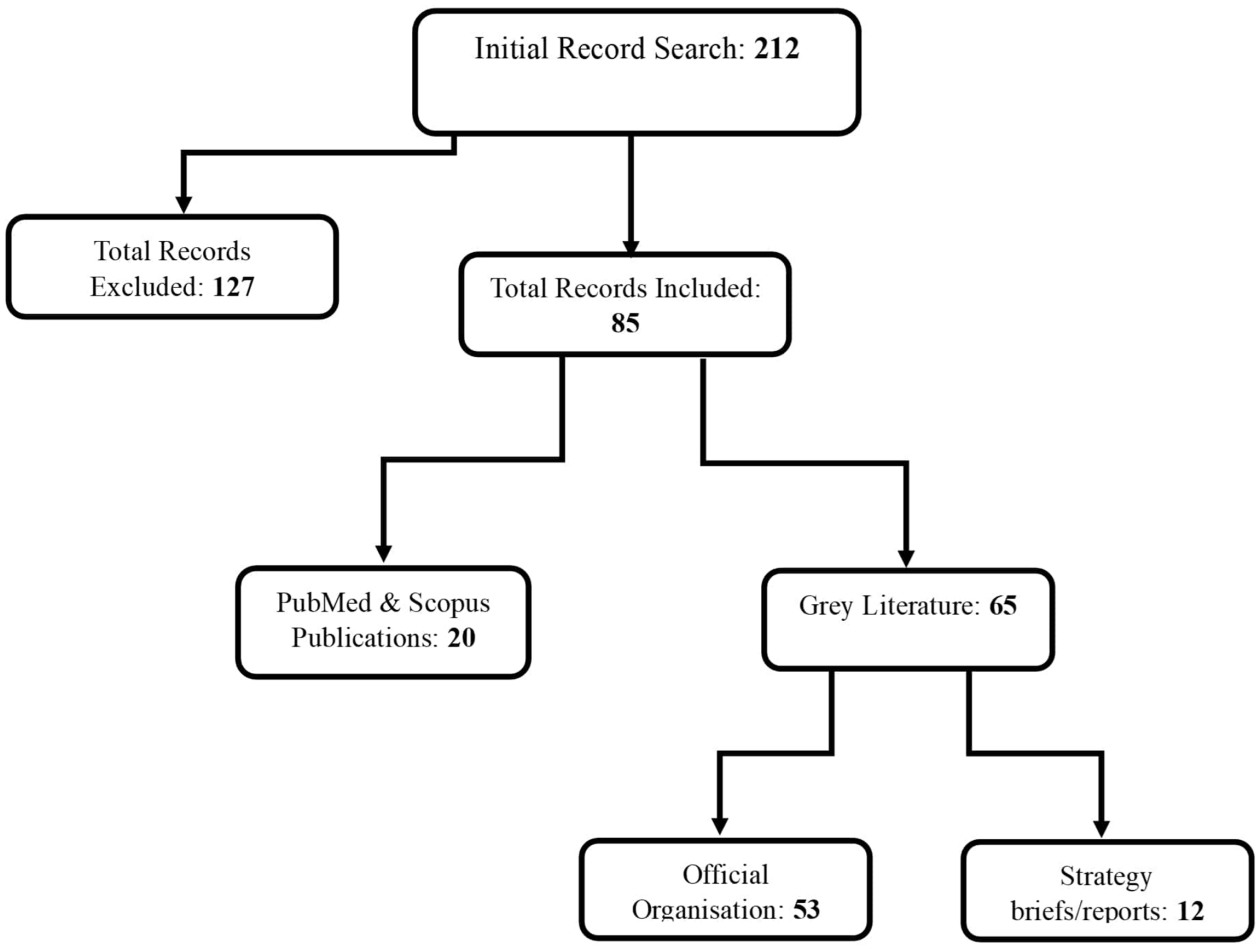
Flow chart illustrating a detailed breakdown of all publications and grey literature records that met the inclusion criteria for this paper, and the total number of records excluded.

**Figure 2:**
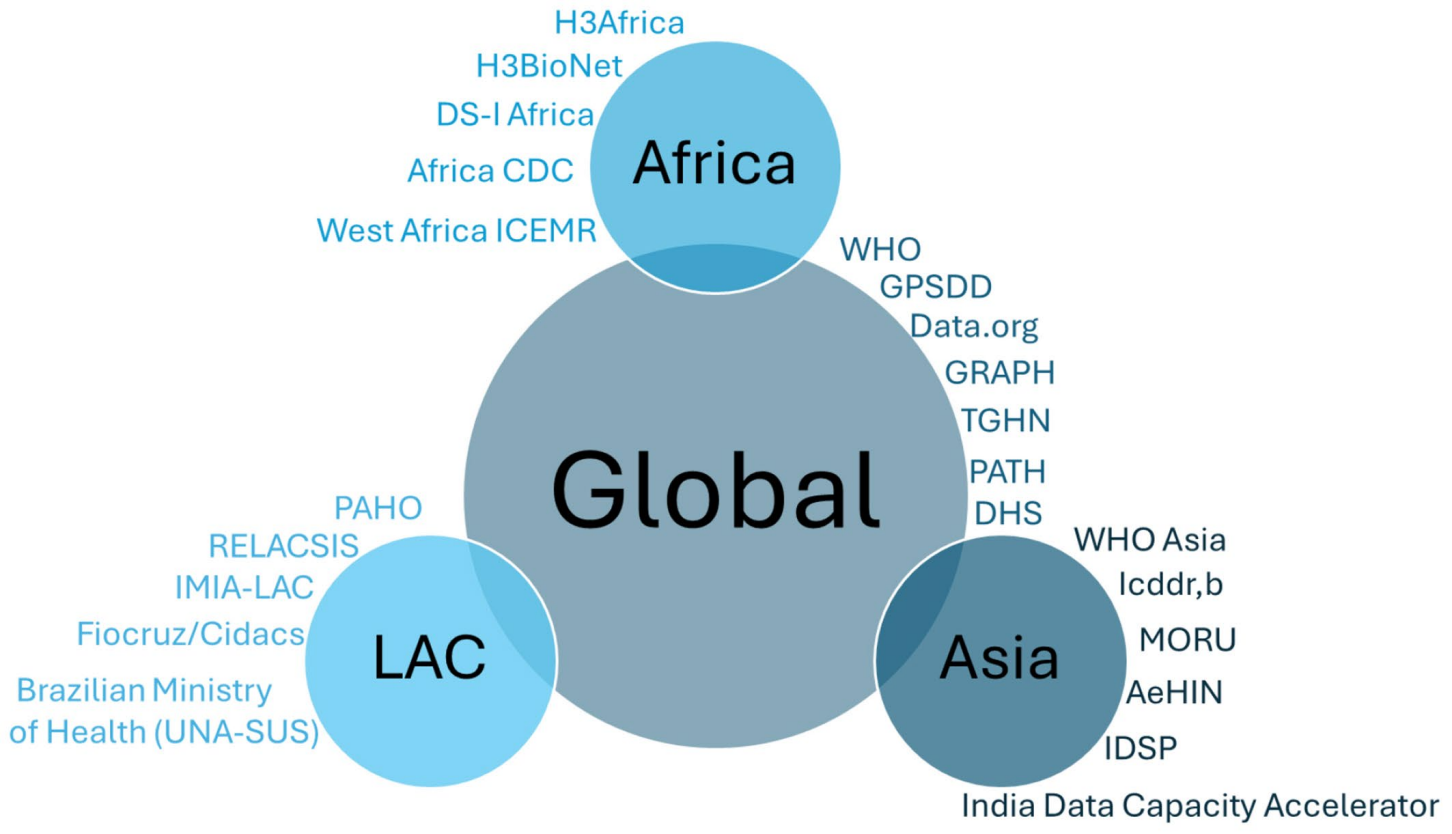
Summary of organisations included in this review that are engaged in health data science capacity building activities.

Our search strategy encompassed a total of 212 records across PubMed, Scopus, and grey literature. In total, 82 records including both publications and grey literature, met the inclusion criteria, while 130 were excluded. Among the included records, 17 were retrieved from PubMed and Scopus, with the majority overlapping between the databases, while 65 were sourced from grey literature, comprising 58 from official websites and 7 from strategy/policy reports. A breakdown of these records is presented in Figure 1. Drawing from the identified records, we have organised the findings on health data science capacity building initiatives into the following sections. Firstly, we present initiatives with a global scope, emphasising those active across multiple low-and middle-income countries (LMICs) or cross-regional, totalling 25 records. Next, we explore regional initiatives specifically dedicated to health data capacity building in Africa, which comprises 20 records, followed by those initiatives in Asia, and subsequently in LAC, both with 17 and 20 records, respectively. Figure 2 presents a graphic illustration of all the major organisations that are actively involved in data science capacity building to address global and regional health challenges.

The findings from the identified records highlight a broad spectrum of priority health areas where organisations have integrated data science capacity building efforts. However, only a minority have implemented detailed data science capacity-building strategies within their health-focused initiatives: in particular, details on coordination, monitoring mechanisms and sustainability plans are not well-documented. This suggests a fragmented approach to data capacity building, rather than a coordinated one. A summary of all health data science capacity building initiatives covered in this review can be found in Supplementary Table S2.

### 3.1 Global and cross-regional data science capacity building initiatives for health research

#### 3.1.1 The Global Partnership for Sustainable Development Data (GPSDD)

The GPSDD, established in 2015, has played a transformative role in advancing data science in LMICs (28). Operating across 34 countries and involving governments, international organisations, civil society, academia and the private sector, GPSDD aims to foster data collaboration, innovation and enhance data ecosystems and bridge the data gap in LMICs by sharing best practice, building technical skills, and data literacy. Aligned with the United Nations’ Sustainable Development Goals (SDGs), GPSDD serves as a robust platform addressing data-related challenges and promoting evidence-based decision-making to contribute to the achievement of SDGs (29). In its six-year strategy (2024–2030), GPSDD prioritises capacity building in key areas of data science and knowledge dissemination at all levels (30). Emphasising data interoperability, past strategies have incorporated standards and facilitation of peer-to-peer learning (30). GPSDD is also committed to enhancing knowledge sharing within its network, promoting advanced techniques, including big data analytics, and fostering data science capacity through exemplar initiatives, such as the “Food Systems Transformation” programme, focused on food security and healthy diets for all. This programme focuses on developing and accessing data, tools, methods and analysis to accelerate and optimise food system transformation at the country level (31). GPSDD’s capacity-strengthening strategy includes leveraging their network of over 700 partner organisations, by acting as a capacity learning convenor to deliver practical skills and knowledge-sharing across the network. This encourages a diverse range of learning opportunities, including peer exchanges, workshops, online training and an in-depth fellowship programme, with a focus on scaling these efforts to reach more partners and strengthen local systems. Such examples include collaborations such as the Climate and Health Capacity Accelerator Network, which equips emerging data professionals with interdisciplinary skills through partnerships with local and international institutions. GPSDD ensures sustainability and impact by aligning strategies at national, regional, and global levels, advocating for data ecosystems that reflect shared values, brokering partnerships, and coordinating across agencies and organisations to align data governance practices and minimise confusion, duplication, and divergence. Additionally, the organisation builds political coalitions, mobilises practical skills and knowledge, and fosters national and regional partnerships to promote inclusive and accountable data use.

#### 3.1.2 World Health Organization (WHO)

The WHO, established in 1948 and functioning as a specialised agency within the United Nations, plays a pivotal role in the supervision of international public health. WHO is at the forefront of using emerging digital technologies to drive health innovation globally and is implementing a strategy for digital health transformation (32).

The strategy for transforming digital health involves securing additional resources and enhancing data science capabilities across a multidisciplinary spectrum. This encompasses fields that range from computer science to management of health science and care delivery, depending on the specific context and applications (32). WHO’s Capacity Building for Digital Health initiative is designed to cater to a broad spectrum of professionals across various domains, including healthcare workers and information and communication technology professionals, with a specific focus on enhancing their expertise in digital health (32). The initiative aims to empower these professionals with knowledge related to digital health technologies and systems, whilst also equipping them with the skills to develop tools for health data management.

WHO’s data-focused capacity strengthening is further reflected in the creation of its Division of Data, Analytics and Delivery of Impact (DDI) that focuses on best data science practices for the delivery of reliable results, serving as a cornerstone for driving evidence-based policy reforms and tracking their impact (33). Furthermore, WHO has established the Health Emergency and Disaster Risk Management Research Network (Health EDRM RN), which aims to facilitate international and cross-regional knowledge exchange and serve as a global platform for sharing up-to-date evidence and comprehensive information on Health Emergency and Disaster Risk Management (Health EDRM). It provides crucial knowledge and data for policymakers, researchers, practitioners and the broader community, thereby accelerating the development of evidence-informed policies and programmes (34, 35). Key activities include developing and sharing guidance documents, such as “The WHO Guidance on Research Methods for Health Emergency and Disaster Risk Management,” online learning materials, joint webinars, and building regional and professional partnerships. Key milestones are monitored and discussed at the Core Group Meeting. Another noteworthy example of data science capacity building involves the reinforcement of pandemic and epidemic intelligence capabilities across the globe, through the development of the WHO hub for Pandemic and Epidemic Intelligence. The hub aspires to mitigate the effects of pandemic and epidemic risks through enabling collaborative surveillance between nations and communities by offering technical guidance and training on access to scientific data, capabilities and decision-support tools, facilitated through collaboration with national public health institutes and other strategic partners (36, 37). It connects data, solutions and communities of practice globally, fostering an environment for multidisciplinary collaborative intelligence. The hub also strengthens capabilities for forecasting, detection and assessment of health risks through advisory services, training and capacity-building activities, including tailored public health intelligence training modules, technical workshops and hackathons. Key achievements, impact metrics and project highlights of the hub are collated and available in annual review reports of this programme. Furthermore, the development of tools and toolkits by the World Health Organisation, such as the WHO STEPS survey, represents a significant advancement in health data research capacity building, enabling countries worldwide to systematically collect and analyse data on non-communicable disease (NCD) risk factors (38).

WHO adopts a holistic strategy spanning digital health transformation, pandemic intelligence and broader health data capacity building. Leveraging its global network, WHO fosters international collaboration and knowledge exchange, amplifying the reach and effectiveness of its programmes. Through initiatives like the Health EDRM RN and the WHO hub for Pandemic and Epidemic Intelligence, practical training, resources and tools are provided to empower health professionals in tackling real-world health challenges. WHO also ensures sustainability of its initiatives through rigorous monitoring, annual reviews and the development of widely disseminated guidance documents and online learning materials.

#### 3.1.3 Data.org

Data.org is a prominent organisation founded in 2020 through a collaboration between the Mastercard Center for Inclusive Growth and The Rockefeller Foundation (39). It serves as a platform for building partnerships in the realm of data, with the overarching goal of increasing data utilisation to address pressing global challenges and improve the lives of millions worldwide (39). This mission places a significant emphasis on LMICs. A central element of the initiatives that Data.org supports is the strengthening of data science capabilities. The initiatives focus on promoting inclusive prosperity (40), addressing climate change and combating pandemics; and examples of data science capacity building in public health include the Capacity Accelerator Network (CAN) and the Global Epidemic Response of the Future (Epiverse) (41–43).

CAN’s main purpose is to build data capacity accelerators in LMICs that enable the training of the next generation of data practitioners by 2032, providing the interdisciplinary skills needed for working at the intersection of climate and health (42). Epiverse, a global collaborative programme supported by Wellcome and The Rockefeller Foundation, is dedicated to creating a reliable data analysis ecosystem for monitoring infectious disease outbreaks and better management of future epidemics and pandemics (43). This ecosystem aims to proactively address future public health crises by offering a standardised and readily accessible set of epidemiological software tools. The Epiverse initiative is committed to advancing the field of epidemiology through its three fundamental pillars: Epiverse TRACE, Epiverse BUILD and Epiverse Connect, all designed to develop innovative open access software tools, foster a community that drives positive change, and promote co-creation and collaboration to enhance epidemiological practices and research (43).

A notable illustration of the Epiverse initiative’s focus on enhancing data science capabilities is the regional initiative known as Epiverse TRACE-LAC. This initiative focuses on strengthening the data science ecosystem for epidemics within the LAC region (43). It has achieved substantial progress by mapping stakeholders and identifying obstacles to accessing tools and training. One of its key priorities is to strengthen regional data analytics capabilities through diverse educational strategies, encompassing both in-person and e-learning approaches and targeting a broad audience, including public health professionals, students and science, technology, engineering and mathematics (STEM) researchers (43).

Data.org is committed to supporting initiatives that have a holistic approach to data science capacity building through strengthening regional data ecosystems and promoting collaborative learning among diverse stakeholders. This not only builds essential skills in data science but cultivates a community-driven environment that enhances epidemiological practices worldwide. However, it is currently unclear how these initiatives monitor their activities and impact, highlighting the importance of having a standardised framework with clear monitoring mechanisms to assess effectiveness and long-term impact.

#### 3.1.4 PATH

PATH is an international non-profit organisation established in 1977 and based in Seattle, USA, with a mission to advance health equity worldwide through partnerships with funders, public institutions and the private sector. It aims to address the most urgent health challenges facing the world, particularly in LMICs (44). PATH actively supports a wide range of programmes addressing global health challenges, covering topics that include Digital Health and Data (45). The Digital Health and Data programme seeks to support LMICs in the development and advancement of digital health transformation and data use, through strengthening of and investment in digital health leadership, capacity and digital health systems (45). Central to this programme is data capacity building, which aims to elevate the skill sets of a diverse range of professionals, including healthcare practitioners, data analysts, technology experts and researchers (45). This is achieved through rigorous training, mentorship and the creation of collaborative environments in the data science domain, particularly focused on emerging technologies. The ultimate goal is to empower individuals to effectively harness digital innovations, thereby improving healthcare delivery and the efficiency of healthcare systems and contributing to better health outcomes in communities worldwide (45). PATH works closely with global funders to align funding and achieve harmonisation, based on global, national and sub-national priorities and needs. This ensures that health data science capacity strengthening meets the specific needs of researchers and health practitioners and enables them to create meaningful impact. Moreover, PATH showcases the impact of the initiatives it supports through their annual review reports as well as by selective case studies showcased on their website. However, it is unclear whether PATH adheres to a structured implementation framework or employs specific mechanisms for monitoring its activities.

#### 3.1.5 The Global Research and Analyses for Public Health (GRAPH) Network

The Global Research and Analyses for Public Health (GRAPH) Network, established through a collaboration between the WHO African Regional Office (WHO AFRO) and the University of Geneva - ISG, is a multidisciplinary community aimed at enhancing data management, analysis, and reporting capabilities (46). Initially focused on supporting AFRO member states during the COVID-19 pandemic, GRAPH develops advanced tools and training materials to bolster disease surveillance and public health research globally. The network includes health professionals, universities, NGOs, and international organisations from over 30 countries, emphasising the importance of multidisciplinary collaboration in public health (46).

To address the need for skilled public health data analysts in LMICs, the GRAPH network is developing an open-enrolment training programme^1^ that leverages modern digital pedagogy for scalable and affordable training. The programme features multi-modal content delivery, interactive code tutorials, user progress tracking through rigorous assessments and certificates, community support through discussion groups, project-based assignments and connection to the open-source EpiGraphHub platform, which continuously aggregates, cleans, and harmonises data to produce actionable reports for health authorities (47). This platform guarantees access to up-to-date health datasets and integrates with local health management systems like DHIS2 and REDCap. Integrated into the GRAPH Network Training Program, EpiGraphHub enables health professionals to perform global-scale data analysis in familiar R and Python environments (47). As an open-source tool, EpiGraphHub can be deployed locally by any country, fostering closer collaborations between countries and technical agencies in surveillance and outbreak analyses (47). The GRAPH Network showcases the impact of their data science capacity strengthening through journal publications who also make available on their website.

#### 3.1.6 The Demographic and Health Surveys Program (DHS)

The DHS Program, founded by the United States Agency of International Development, is a global initiative that collects and analyses data on population, health, and nutrition in LMICs. Since its inception in 1974, it has conducted over 320 Demographic and Health Surveys (DHS) across 90 countries, yielding valuable insights into critical health issues (48). This programme offers free access to survey datasets, reports and other resources, along with tools for analysing and visualising data, and provides support to researchers and policymakers in analysing and interpreting DHS data for their specific needs. Through the DHS Learning hub, the programme supports data science capacity building through open courses, workshops and tutorials in survey data analysis (49). The Program’s capacity-strengthening strategy prioritises achieving targeted results across the survey process continuum, emphasising skills development through evidence-based best practices and blended learning methods. Collaboration with host-country partners ensures local ownership, whilst rigorous evaluation processes foster continuous improvement and learning. In-country activities include regional and national training options to support these efforts. Moreover, the DHS Program conducts capacity assessments to identify strengths and gaps, focusing on technical capabilities and responsiveness to data users’ needs. Based on these assessments, tailored country-specific capacity strengthening plans are developed, utilising options such as online training, virtual coaching, and workshops to enhance individual and institutional skills. They employ refined learning approaches, including participatory methods and competency-based training, supported by mentoring and online resources, to meet the specific needs of DHS countries across the survey continuum.

#### 3.1.7 The Global Health Network (TGHN)

TGHN operates a network of knowledge sharing hubs to facilitate health research in challenging environments, fostering the exchange of knowledge, methods and research findings across diseases, regions and organisations (50). These hubs also offer regional and online training, resources, tools, templates and professional development opportunities to enhance skills and careers, ultimately aiming to drive evidence-based practice. A cornerstone of TGHN is its digital Training Centre, offering free online courses and extensive face-to-face activities to cultivate research skills. Amid the challenges of 2020, TGHN successfully pivoted to online formats for webinars and workshops, ensuring continuity in training delivery. Additionally, TGHN implements a Professional Development Scheme to monitor skills, track progress, and identify knowledge gaps, further supporting the ongoing development of researchers worldwide and documents metrics along with impact in its annual review report.

Several digital knowledge hubs within TGHN provide tools and resources relevant to health data scientists. The European and Developing Countries Clinical Trials Partnership (EDCTP) Hub offers a data management portal and sharing toolkit (51), and the Global Health Data Science Hub (GHDSH) (52) serves as an open-access knowledge exchange platform for data scientists in health research. Supported by a global health data science community, the GHDSH aims to share relevant tools, resources, and support, fostering collaboration and knowledge sharing among data scientists. Both hubs feature measurements on their impact pages, such as number of visitors, views and resource downloads. However, establishing a structured framework to track how these training opportunities translate into measurable health or social impacts would provide valuable insights and enhance effectiveness.

TGHN operates additional knowledge exchange hubs in LAC (53), Africa (54) and Asia (55), serving as valuable regional resources for health data scientists and researchers.

### 3.2 Data science capacity building initiatives for health research in Africa

#### 3.2.1 West Africa International Center of Excellence for Malaria Research (ICEMR)

The establishment of the West Africa ICEMR represents a significant milestone within the broader ICEMR Network, initiated by the National Institute of Allergy and Infectious Diseases (NIAID) in 2010 (56). This network aims to address research barriers hindering effective malaria control across 20 countries affected by the disease. The West Africa ICEMR focuses on malaria control, treatment and prevention within the Sub-Saharan West African regions (56).

A major achievement of the West Africa ICEMR initiative is the development of a robust and sustainable data collection management system (DCMS) that links study sites in Mali, Senegal, and The Gambia (57). Capacity building has been a critical aspect of ensuring the DCMS’s success, with extensive training provided in data management, geographic information systems, statistical analysis, and scientific paper writing for principal investigators and data managers. This robust training has strengthened the West Africa ICEMR facilitating future research projects and enhancing local research leadership (57).

The initiative has significantly contributed to the research career development of numerous junior investigators. Sponsored research has offered internship and thesis opportunities for graduate students, addressing malaria prevention and control research questions. Additionally, the West Africa Center of Excellence in Bioinformatics Training provides short-and long-term training in bioinformatics and data science, along with funding for independent pilot projects for students and postdoctoral researchers (58).

Operational and implementation research training workshops are regularly organised with National Malaria Control Programs (NMCP) and partners to identify research questions that can improve control implementation. These workshops increase awareness among malaria control programme managers and district healthcare officers on the latest research findings, whilst also generating research questions for graduate students’ thesis research and career development (58).

The West Africa ICEMR has maximised engagement amongst NMCP, local, national and international organisations, and research communities. This collaboration empowers local researchers and builds the next generation of malaria researchers, facilitating the uptake of research findings into policy and programme activities. Whilst each study reports its findings and capacity-building efforts through traditional publications, a coordinated system for reporting these activities is lacking.

In summary, the West Africa ICEMR focuses on capacity building through integrated research studies rather than separate activities and funding. This integrated approach arguably supports a sustainable framework, though the success and extent of these capacity-building efforts remain unclear beyond traditional publications.

#### 3.2.2 H3Africa and H3BioNet Initiatives

The multi-country Human Heredity and Health in Africa (H3Africa) consortium, which was established in 2012 and funded by the USA’s National Institute of Health (NIH) and Wellcome, is dedicated to empowering African researchers to excel in genomic sciences, fostering robust collaborations among scientists across the continent, and generating valuable data to enhance both African and global health (59, 60). This pioneering initiative has laid the groundwork for significant advancements in genomic studies on the continent. To ensure the continuity of this critical work, H3Africa places significant emphasis on capacity building, focusing on various essential elements such as ethical, legal and social implications research, bioinformatics training, biobanking capacity, and coordination and networking.

The consortium’s approach to capacity development spans short, medium and long-term priorities. It encompasses human capacity building, provision of necessary hardware and resources, and the development of technology platforms. H3Africa targets interdisciplinary researchers, including bioinformaticians, genomics researchers, clinical scientists and ethics experts, with training formats that range from short courses and internships to formal curricula, degree programmes and online training (59, 60). In the short term, H3Africa focuses on bioinformatics and biostatistics training. Long term, the goal is to develop faculty trained in genomics and bioinformatics, produce MSc and PhD graduates, and train technicians. The initiative also invests in essential computing infrastructure, provides access to existing computing resources and tools, and develops new tools as needed (59, 60). Technology platforms are either utilised or developed within Africa, ensuring setup, quality control, maintenance and long-term sustainability. Where necessary, outsourcing to commercial companies or collaborators abroad is considered. H3Africa comprises approximately 500 members across 26 different projects, networks, and sub-nodes in 32 countries, supported by over 10 working groups, an Informatics Network, three Bio-repositories, and a Data and Biospecimen Access Committee. The consortium is overseen by a Steering Committee with guidance from an Independent Expert Committee. To maintain cohesion and coordination within this complex network, the H3Africa Administrative Coordinating Centre (H3ACC) plays a pivotal role. The H3ACC ensures quality administration, provides fiscal and logistical support for consortium activities, oversees cross-consortium collaborations and resources, facilitates the pooling of training resources and career-tracking, monitors and evaluates progress, and engages stakeholders to increase awareness and support for genomics research (61).

One of the direct outcomes of H3Africa’s vision and support has been the establishment of H3Africa Bioinformatics Network (H3ABioNet), a Pan-African Bioinformatics Network sponsored jointly by H3Africa and the NIH (62, 63). The primary aim of H3ABioNet is to fortify the bioinformatics landscape, foster the development of essential skills and tools, and promote knowledge dissemination across Africa. With its extensive network comprising 27 nodes spread across 16 African countries, H3ABioNet has proven instrumental in providing sustainable and specialised bioinformatic skills training to diverse audiences, significantly enhancing the research capabilities of African scientists and driving the progress of genomic research within the continent (62, 63).

H3ABioNet has adopted a multi-layered approach to delivering sustainable bioinformatics training across the continent. As of May 2021, their efforts yielded substantial success, with an impressive participation of 4,466 trainees from diverse science backgrounds on one or more of their courses. Since its inception, the initiative has fostered essential bioinformatics and computational skills among bioinformatics analysts and engineers, developed guidelines, and supported the establishment of introductory and specialised intermediate programmes to enhance user competencies. A noteworthy accomplishment is the establishment of novel bioinformatics degree programmes at resource-constrained institutions, exemplified by the Master of Science (MSc) in Bioinformatics established in 2015, in collaboration with the University of Science of Technical and Technology De Bamako (USTTB). To date, this programme is one of only 13 specialised courses offered across 7 African countries (64, 65). Furthermore, H3ABioNet has established the Introduction to Bioinformatics Training Course (IBT) with the support of the Fogarty International Centre (FIC) and the “H3ABioNet 16S rRNA Microbiome Intermediate Bioinformatics Training” course (16S course), offering additional research capacity in genomics for beginner and intermediate-level researchers (64, 65). The success of the IBT and 16S courses lies in their innovative mixed-model course delivery approach, combining distance learning through online lectures, a Learning Management System, Open Educational Resources (OER), and face-to-face learning in local classrooms with support staff (66, 67). This unique combination overcomes the challenges associated with traditional instructional methods, providing students with flexible access to course materials, enriched learning resources, and personalised guidance (66, 67). H3ABioNet has effectively addressed some of the diverse computational challenges faced by researchers in the field by developing four robust workflows that can be executed in heterogeneous computing environments, ensuring a seamless and consistent software environment and promoting ease of use and reproducibility across various computing systems and platforms (68, 69). Moreover, to further strengthen bioinformatics capacity development, H3ABioNet established an online bioinformatics help desk, known as the H3A-HD, offering ongoing support through technical experts across diverse bioinformatics sub-disciplines (70).

The enduring success of this programme can also be attributed to the way in which it fosters connectivity and collaboration amongst participants across the continent and beyond. This is achieved through use of structured forums and real-time chatting tools, made possible by leveraging their advanced Learning Management System, Vula (68). H3ABioNet is actively expanding and playing a leading role in building bioinformatics capacity in Africa, and their efforts are driving significant progress in genomics and related health research, fostering valuable advancements in the field.

#### 3.2.3 Data Science for Health Discovery and Innovation in Africa (DS-I Africa)

The DS-I Africa programme is an ambitious initiative designed to leverage data science and advanced technologies to drive health discovery and innovation across the African continent (71). This programme is driven by the mission of cultivating essential data science expertise among African scientists and researchers, empowering them to harness the power of data science in addressing critical health challenges. One of the primary objectives of the DS-I Africa programme is to foster robust networks of African investigators, promoting collaboration and knowledge exchange to enhance health research and development efforts in Africa.

To achieve this, DS-I Africa incorporates various components to enhance data science capacity building across the continent. Training programmes provide trainees with a thorough understanding of research design, methods and analytic techniques, while encouraging interdisciplinary experience and innovative solutions. A coordinating centre, serving as the organisational backbone, oversees common activities. Research hubs, recognised as centres of excellence in data science, focus on creating scalable, population-relevant and affordable solutions to improve health outcomes in Africa. The initiative also features an open data science platform called eLwazi, which facilitates data-sharing gateways for existing and new data generated by research hubs. Moreover, DS-I Africa supports addressing ethical, legal, and social aspects of research, covering data privacy, ownership, cybersecurity, and the sensitive use of geospatial information. Together, these components of DS-I Africa foster collaboration, knowledge sharing, and the development of impactful global tools and applications (71, 72).

D-SINE Africa’s Capacity Building Core (CBC) specifically addresses recognised barriers to the development and success of investigators in Sub-Saharan Africa (SSA). The CBC catalyses the conduct of high-quality, applied data science research to improve community health in Africa, including addressing the pervasive problem of injury. The CBC supports the capacity of D-SINE Africa’s cores, projects, and partners to administer, manage, conduct and disseminate high-quality data science research designed to enhance innovation. Additionally, the CBC integrates with the capacity-building efforts of the DS-I Africa Consortium, including the Research Training Programs (73).

The CBC curates and makes available a “Data Science Methods Toolkit” to mentor and enable emerging African scientists to incorporate data science methodologies into research projects, propelling junior scientists towards independence. A Seed Grant Program is implemented to launch the careers of junior African scientists working at the intersection of injury, surgery, and equity. Furthermore, the CBC creates an enabling administrative and financial environment at D-SINE Africa institutions to successfully administer and manage D-SINE Africa and facilitate the completion of its goals (73).

Through these multilayered efforts to data science capacity building, DS-I Africa ensures the sustainability and impact of its initiatives, driving significant progress in health discovery and innovation across Africa. The programme’s integrated approach to training and capacity building via the CBC, provides essential skills and financial support to junior scientists. Its extensive training programmes promote interdisciplinary collaboration and innovation, whilst a coordinating centre and research hubs foster efficient knowledge exchange and resource sharing. Moreover, the commitment of DS-I Africa in addressing ethical, legal, and social aspects of research builds trust and accountability, essential for long-term success. Moreover, the programme’s sustainability is further reinforced by diverse international funding and facilitated by an enabling administrative and financial environment, as well as through the adoption of rigorous monitoring and evaluation processes to ensure continuous improvement and effectiveness in achieving its mission.

#### 3.2.4 Africa Centres for Disease Control and Prevention (Africa CDC)

Africa CDC, established in 2016, is a specialised public health agency operating under the African Union. Africa CDC is dedicated to enhancing the collective health resilience of member states by providing strategic support and technical expertise (74). This includes strengthening the capacity of African public health institutions to effectively identify, monitor and respond to emerging disease threats (74).

Africa CDC emphasises data capacity building through their digital transformation strategy which centres around advancing public health across the continent by leveraging technological advancement (75). This strategy supports the analysis of existing human capital to identify capacity gaps, launching a Health Informatics Fellowship, providing online training, and developing a Pan-African Health Informatics Network. It also involves organising the African Health Tech Summit, defining digital competency frameworks and job classifications, and designing curricula for digital health, ensuring these are institutionalised at the member state level, empowering African researchers whilst also ensuring a more robust response to public health emergencies (75).

Moreover, CDC Africa has placed great importance on the development of a robust Monitoring, Evaluation, and Learning (MEL) framework for the digital transformation strategy. It will establish clear goals, outcomes and indicators to measure progress and impact, as well as ensure alignment with partners and enable informed decision-making to enhance the strategy’s impact and effectiveness over time (75).

A further noteworthy endeavour in capacity building is the Africa CDC Institute of Pathogen Genomics, which focuses on enhancing disease surveillance and fostering public health collaborations through integrated, cross-continent laboratory networks. These networks will be equipped with the necessary tools, human resources and data infrastructure to effectively harness critical genomic sequencing technologies. The Institute aims to strengthen technical capacity and knowledge sharing through enhancing laboratory systems and enhance genomic surveillance capacity, with priorities including expanding molecular diagnosis capacity, implementing high-priority use cases, sharing genomic data, and creating a sustainability mechanism through advocacy, policies, governance, and funding by establishing a Pan-African network, comprising 20 national public health institutes across the continent (76). Additionally, through the development of the Next Generation Sequencing (NGS) Academy, the Institute of Pathogen Genomics strives to offer further training opportunities, including specialised curriculum development in bioinformatic techniques to advance research in pathogen NGS (76). In summary, Africa CDC’s integrated approach to capacity building, through its digital transformation strategy and robust partnerships, aims to create a sustainable framework that enhances Africa’s ability to respond to public health emergencies and advance genomic research.

### 3.3 Data science capacity building initiatives for health research in Asia

#### 3.3.1 World Health Organization: Regional Office for South-East Asia

The World Health Organization’s Regional Office for South-East Asia has developed the “Regional Strategic Roadmap on Health Security and Health System Resilience for Emergencies 2023–2027” (77). This roadmap aims to enhance national health security and resilience during emergencies by addressing key elements such as leadership, finance, information, surveillance, intelligence, public health systems, community engagement and supporting infrastructure.

A central focus of the roadmap is capacity building, particularly in training healthcare professionals, researchers and policymakers in emerging technologies critical for emergency responses and safety. This involves developing systems for collecting and analysing big data, creating predictive models, and integrating solutions into healthcare systems. By fostering data science capacity, WHO aims to improve public health in Southeast and East Asia. The approach includes ensuring efficient and adaptable surveillance systems for early warning and rapid response, strengthening genomic surveillance, and enhancing data management capacities. WHO also emphasises the effective use of digital and information technology for surveillance and information management (77).

Sustainability and impact are ensured through a robust monitoring and evaluation framework aligned with the International Health Regulations (IHR). This framework, developed in consultation with Member States, includes both hardware (infrastructure, equipment, workforce) and software (trust, morale, innovation support) indicators to assess preparedness and response efficiency (78). By continuously engaging with Member States and incorporating learning systems, the WHO aims to refine policies and implementation strategies, ensuring a comprehensive and effective health security response across the region.

Despite these efforts, the specifics of how the capacity building activities will be implemented remain unclear. Given the regional disparities in infrastructure and training capacity, it is essential for the roadmap to provide more transparency and clarity on how these activities will be coordinated.

#### 3.3.2 Mahidol Oxford Tropical Medicine Research Unit (MORU)- Tropical Health Network

The MORU Tropical Health Network, established in 1979 through collaboration between Mahidol University and the University of Oxford and funded by the Wellcome Trust, operates satellite units including COMRU, LOMWRU, MOCRU, and SMRU. It addresses a spectrum of health challenges in Asia, spanning malaria, maternal and child health, infections, critical illness, medicine quality, statistics, data modelling, bioethics and community engagement, aiming to develop cost-effective interventions (79–81).

A cornerstone of MORU’s strategy is enhancing health data research capabilities. This includes leveraging geospatial data for disease surveillance, conducting data management workshops, and pioneering mathematical and economic modelling through MAEMOD in Bangkok. These efforts support curriculum development at Oxford and Mahidol Universities, fostering expertise in biomedical and health information (82–85). In parallel, the Clinical Trials Support Group (CTSG) within MORU plays a pivotal role in promoting research excellence and compliance. Since 2015, CTSG has facilitated data sharing through regional workshops in Cambodia, Bangkok, Oxford, and Vietnam, leading to the development of the Data Sharing for Health Research course. They have also launched advanced Statistics courses and implemented a toolkit for research consistency and Good Clinical Practice (GCP) training across MORU units (82–85). Looking forward, CTSG continues to expand technical forums and develop new training materials to strengthen research capacity across the MORU network, ensuring sustained impact and innovation in tropical health research.

However, whilst MORU has made significant strides in technical training and capacity building, challenges remain. The approach to data sharing and collaborative workshops, although commendable, could benefit from clearer metrics for impact assessment and long-term sustainability. The effectiveness of these training programmes in translating knowledge into tangible health improvements needs robust monitoring frameworks to ensure continuous adaptation and improvement. Moreover, ensuring that training initiatives reach beyond immediate participants to influence broader healthcare practices in MORU’s network requires ongoing investment in infrastructure and human resources. Evaluation of the long-term impact of these capacity-building efforts will be essential to refine strategies and optimise resource allocation.

#### 3.3.3 International Centre for Diarrhoeal Disease Research, Bangladesh (icddr,b)

The icddr,b, a major international research institute in Dhaka, Bangladesh, has been committed to improving public health since its founding in the 1960s. Whilst initially focusing on diarrhoeal diseases, icddr,b has expanded its research to include a broader range of health issues. Its mission involves pioneering scientific research and informing health policy, grounded in understanding local contexts, developing practical solutions and generating broadly applicable evidence. The institution has significantly impacted public health in Bangladesh and globally (86).

Capacity building is integral to icddr,b’s identity, with education as a cornerstone. Since 1978, icddr,b has trained over 68,000 participants from 87 countries, reflecting its long-term commitment. It offers technical training, internships, and academic programmes for public health practitioners, healthcare professionals, and researchers. Collaborating with partner universities and institutions, icddr,b provides training in epidemiology, biostatistics, research ethics and specific disease areas. Its 2023–2027 strategic plan emphasises mentoring and communities of practice for young scientists, supported by a Technical Training Unit, knowledge repository and online training to enhance accessibility. Annually, 300 students undertake internships (87).

Fostering data science capacity is a key focus. The Centre for Data Excellence aims to build local health manager capacity for health emergency decision-making and link to global data centres (88). The Global Health Network Asia, hosted by icddr,b, enhances data science capacity in Bangladesh and Nepal through country hubs, Research and Data Clubs, and regional networks. This approach leverages data and machine learning to advance public health research.

However, specific implementation strategies for these capacity-building activities need to be clearer. Given regional disparities in infrastructure and training capacity, the roadmap must provide transparency and coordination details. Establishing clear strategies and transparent coordination is crucial for ensuring sustainability and impact.

#### 3.3.4 The Asia Pacific Malaria Elimination Network (APMEN)

Established in 2008, the Asia Pacific Malaria Elimination Network (APMEN) is a collaborative initiative aimed at eradicating malaria from the Asia Pacific region by 2030 (89, 90). Serving as a pivotal platform for countries and stakeholders, APMEN focuses on enhancing malaria management and control strategies, including vector control, surveillance, and addressing outbreaks of Plasmodium vivax. Beyond these core activities, APMEN places significant emphasis on building data science capacity as a cornerstone of its strategy.

APMEN’s approach includes innovative capacity-building initiatives such as cross-regional workshops and tailored training programmes. These workshops facilitate knowledge exchange across multiple countries, focusing on advanced data quality dimensions, GIS mapping and other critical aspects of malaria surveillance. Tailored training programmes are designed based on comprehensive needs assessments, addressing specific skills in malaria vector surveillance, case investigation, GIS mapping and data quality assurance throughout the surveillance data pipeline. Moreover, APMEN provides targeted technical support to countries demonstrating interest, aiming to enhance the quality of Reactive Case Detection (RACD) and foci investigations tailored to local contexts (89, 90).

Sustainability and impact are central to APMEN’s initiatives. The network fosters long-term partnerships with entities like the Roll Back Malaria Surveillance, Monitoring, and Evaluation Reference Group (SMERG SP&DQ) Committee, ensuring continuous knowledge exchange and support. Outputs from training sessions are widely disseminated to strengthen linkages between research institutions, National Malaria Control Programmes (NMCPs), potential donors and other stakeholders. This dissemination enhances the visibility and applicability of research findings, contributing to sustained support for malaria elimination efforts across the region. Moreover, APMEN has formed a collaborative partnership with the Asia Pacific Leaders Malaria Alliance (APLMA), aiming to enhance the regional response to malaria by strengthening elimination efforts through political advocacy and leveraging APLMA’s and APMEN’s multisectoral technical expertise (89, 90). Within this collaborative partnership, a Surveillance Response Working Group (SRWG) has played a pivotal role. The SRWG meets annually to discuss malaria elimination challenges and highlight key priorities and has placed particular emphasis on data capacity enhancement through identifying research gaps in malaria research and committing to address these appropriately. The SRWG is also committed to recognising training needs, especially in areas such as data quality, integration and technology, leading to the comprehensive development of capacity building initiatives (89, 90).

Whilst APMEN’s approach appears robust, challenges such as varying infrastructure and technological capabilities among member countries may affect implementation. The effectiveness of these initiatives hinges on the adaptability of training modules to diverse settings and the sustained engagement of stakeholders beyond workshop settings.

#### 3.3.5 India Data Capacity Accelerator

The India Data Capacity Accelerator programme, an integral part of the Capacity Accelerator Network (CAN) under Data.org, represents a significant collaboration aimed at accelerating the use of data for social impact (DSI) in LMICs (88). Supported by Wellcome and in partnership with J-PAL, the initiative is conducted in collaboration with three Indian universities: Ashoka University, the Birla Institute of Technology and Science, Pilani (BITS Pilani), and the Indraprastha Institute of Information Technology (IIIT) in Delhi.

The programme is designed to equip data researchers and practitioners with essential skills to address climate-related health challenges exacerbated by the ongoing climate crisis. In addition to providing multidisciplinary academic training, the India Data Capacity Accelerator offers fellowship opportunities and internships in partnership with these universities. This initiative also leverages interdisciplinary expertise in climate and health data to foster innovative solutions, thereby making substantial contributions towards mitigating climate-related health issues, not only in India but also globally (88, 91).

The CAN initiative encompasses several key components, including: a 3-month training course on data analysis, stewardship and ethics; the CAN Fellowship; capacity-building workshops; collaborative projects; and strategic partnerships. This comprehensive approach aims to build a robust data ecosystem that empowers communities and drives impactful solutions for sustainable development. The CAN training is meticulously designed to provide learners with a practical and comprehensive introduction to data science, encompassing data collection, analysis, visualisation, machine learning and ethics. It includes live sessions, recorded lectures, assignments, quizzes and case studies, complemented by mentorship from experienced data scientists and instructors. Participants come from various sectors including government agencies, academia, the private sector, and civil society, fostering a diverse learning environment aimed at holistic capacity development.

The programme, now in its first year, lacks clear information on impact tracking and sustainability, and it will be challenging to ascertain the programme’s long-term effectiveness in equipping participants with the necessary skills to drive meaningful change in climate-related health outcomes. Further transparency and clarity in these areas would enhance understanding and confidence in the programme’s ability to achieve its ambitious goals.

#### 3.3.6 Integrated Disease Surveillance Project (IDSP)

The Integrated Disease Surveillance Programme (IDSP), a cornerstone of India’s National Health Mission, exemplifies a strategic approach to enhancing decentralised, IT-enabled disease surveillance across the country (92). Central to its operational framework is a tiered training strategy aimed at equipping healthcare professionals at various administrative levels with essential skills for effective disease monitoring, detection and response.

At the national level, specialised training is provided to Master Trainers, State and District Surveillance Officers, and members of Rapid Response Teams (RRT) at designated institutes. These sessions focus on advanced epidemiology, data analysis, and outbreak management, ensuring robust capabilities among key personnel tasked with oversight and strategic coordination. At the state level, Master Trainers extend their expertise to District Surveillance Officers, Microbiologists, Epidemiologists, Medical Officers, Data Managers and Lab Technicians. This tier emphasises foundational knowledge in disease surveillance principles and epidemiological concepts, essential for accurate data management and local-level response readiness. At the district level, Medical Officers, Community Health Officers, Paramedical Staff, Health Workers and Lab Technicians undergo training aimed at enhancing proficiency in data collection, compilation and reporting protocols. Special attention is given to preparing District Rapid Response Teams for proactive and coordinated responses during disease outbreaks, underscoring their critical role in maintaining public health vigilance (92).

Despite these structured training efforts, challenges persist in ensuring the sustained impact of capacity-building initiatives within the IDSP. Enhanced monitoring and evaluation frameworks are needed to systematically assess the practical application of acquired skills and their contributions to strengthened disease surveillance and response capabilities. Without clear metrics and documented outcomes, it is challenging to gauge the long-term effectiveness of training initiatives and the extent to which they translate into improved disease surveillance and response capabilities at the grassroots level.

### 3.4 Data science capacity building initiatives for health research in Latin America and the Caribbean

#### 3.4.1 Pan American Health Organisation (PAHO)

PAHO is dedicated to building data science capacity across the Americas through strategic initiatives aimed at enhancing health information systems and digital literacy (93). Its “Strengthen Information Systems for Health 2019–2023” action plan underscores the importance of improving data capture and management for informed decision-making, policy development and monitoring (94). This plan includes goals such as building data science capabilities, creating knowledge networks, establishing robust regulatory frameworks, developing sustainable digital health models, and promoting standards for health data.

A key component of PAHO’s capacity-building efforts is its Virtual Campus for Public Health (VCPH) offering self-learning courses, tutoring and country-specific programmes in Spanish, Portugal, English and French (95). This platform provides open and free resources, including health data-related courses on digital health, information systems, and evidence for health action, targeting healthcare practitioners. The virtual campus features individual dashboards for tracking learner progress, and the online campus also has incorporated built in metrics tracking such as user’s location, professions and education status. However, PAHO also acknowledges the need for improved assessment tools to fully measure the impact of its programmes.

Countries in the region face numerous challenges in strengthening their health information systems. These include updating legal frameworks, implementing data-sharing agreements, developing governance models, creating standardised health data dictionaries, and adopting standards and procedures for continuous quality improvement. PAHO sees an opportunity for networked collaboration to address these common challenges, aiming to convert outcomes into public good for the region.

To ensure sustainability and effectiveness, PAHO’s strategy includes monitoring and evaluation aligned with the organisation’s results-based management framework. Progress towards the action plan’s goals will be evaluated through a progress report to PAHO’s Governing Bodies in 2022, with a final report due in 2024.

Overall, PAHO’s efforts represent a robust approach to data science capacity building, addressing critical needs in health information systems and digital literacy. The organisation’s comprehensive, accessible, and multilingual training programmes are commendable. However, continuous refinement of evaluation mechanisms and practical application in the field are essential for translating education into improved health outcomes across the region.

#### 3.4.2 The Latin American and Caribbean Network for Strengthening Health Information Systems (RELACSIS)

The Latin American and Caribbean Network for Strengthening Health Information Systems (RELACSIS), established by PAHO and the United States Agency for International Development (USAID), serves as a community of practice for professionals and technicians who use and produce health information in the Latin American and Caribbean (LAC) region (96). The primary mission of RELACSIS is to enhance the quality of data, diagnostics, and health policies across the region, covering all stages of data gathering and analysis (96).

RELACSIS focuses on providing training in best practices and fostering the development and use of cost-effective, high-impact tools to improve health information systems. Among its offerings are online courses related to data quality, data analysis, and data interpretation, as well as comprehensive training packages on ICD-10 and ICD-11 coding practices (96). These initiatives provide a structured framework and language for the reporting, completion, utilisation and sharing of health information on both national and international fronts.

The network emphasises South–South cooperation, enabling participating countries to share experiences and develop common solutions to common problems. This collaborative approach ensures that best practices and effective tools are disseminated throughout the region, enhancing the overall capacity of health information systems.

RELACSIS’s focus on training with low-cost/high-impact tools is particularly noteworthy. By providing accessible and practical resources, the network ensures that even countries with limited financial resources can benefit from advanced training and improve their health information systems. This approach not only enhances the immediate capabilities of health professionals but also contributes to the long-term sustainability and improvement of health data quality in the region.

However, despite providing online training opportunities and tools, question marks remain on the successful implementation and impact of these initiatives, considering the variability and technological infrastructure across the participating countries, as well as the lack of tracking mechanisms and impact assessment on how these translate into tangible improvements in health information systems and public health outcomes. In addition, whilst the focus on low-cost/high-impact tools is commendable, the long-term sustainability of these efforts depends on continuous funding, political commitment and ongoing capacity building. Overall, there needs to be a stronger emphasis on building local capacity and ownership to ensure that improvements in health information systems are maintained over time.

#### 3.4.3 Health Informatics Association for Latin-America and Caribbean (IMIA-LAC)

The IMIA-LAC, founded by the International Medical Informatics Association (IMIA) in 1983, is a coalition of regional medical informatics societies with the overarching goal of developing and strengthening health informatics in the LAC region (97, 98). IMIA-LAC’s mission encompasses advancing health informatics education for both users and experts by establishing academic programmes in collaboration with regional universities. The association promotes the adoption of health information systems (HIS), emphasising the importance of HIS standards (97, 98). A development plan is underway for Health Informatics Education, aiming to create structured educational pathways and resources in collaboration with universities.

IMIA-LAC has created various working groups focusing on key areas such as bioinformatics, nursing informatics, and informatics and quality in healthcare. These groups facilitate specialised training and the development of best practices within their respective domains. Additionally, IMIA-LAC works to strengthen connections between academia and government, aiming to facilitate impactful and successful transformations in the field (97, 98).

IMIA offers accreditation for Biomedical and Health Informatics programmes at various institutions, ensuring high standards and promoting continuous improvement through a self-assessment process. The association also connects members to the International Academy of Health Sciences Informatics, which disseminates knowledge and best practices, fosters new ideas, and encourages global collaboration and resource sharing. The current focus of IMIA-LAC includes expanding membership and coordination across the LAC region and securing funding for distance learning initiatives. This expansion aims to increase accessibility to health informatics education and foster a broader network of professionals dedicated to advancing health informatics in Latin America and the Caribbean.

Nevertheless, challenges stemming from differing institutional resources and infrastructure among regional universities may impede collaborative efforts. It will be essential to secure adequate funding for distance learning and to broaden membership to ensure sustainability, necessitating support from local governments and international allies. Successful integration of health informatics education into established academic frameworks and its acceptance within healthcare sectors are pivotal, as are rigorous monitoring and evaluation mechanisms for fostering consistent adoption of standards and practices across the region to facilitate continual enhancement and impact evaluation. It is currently unclear how these will be implemented.

#### 3.4.4 Oswaldo Cruz Foundation (Fiocruz)

The Oswaldo Cruz Foundation (Fiocruz) was established in 1900 and covers health research, science and technology, operating as part of Brazil’s Ministry of Health (99). At its core, Fiocruz is dedicated to strengthening the Brazilian public health system and improving the quality of life in the wider region, and has a presence in all 10 states in Brazil, an office in Maputo (Mozambique, Africa) and a laboratory in Antarctica. Operating across various domains, Fiocruz’s responsibilities include: providing hospital care; conducting research on prevalent diseases in Brazil (such as zika, malaria, tuberculosis, yellow fever, HIV/AIDS and dengue); engaging in comprehensive research and development; and fostering international collaborations for knowledge exchange with over 50 countries worldwide (100). It also offers educational training for the public and for healthcare workers, ranging from technical courses through to PhD programmes and including science communication.

Fiocruz has a strong educational presence through the Fiocruz Virtual Campus, which serves as a hub for knowledge and learning in health education (101). Within this environment, individuals and partner institutions collaborate and share platforms, services and activities. Utilising advanced information, communication and education technologies, learners gain entry to a diverse spectrum of educational resources. With an annual enrolment surpassing 7,000 students, Fiocruz holds a pivotal position as a non-university entity, actively engaged in preparing and empowering human resources to make meaningful contributions to the Brazilian health system (101). Within the broader context of enhancing data science capacity, the Fiocruz Virtual Campus extends its impact through offering workshops and a comprehensive range of courses, which feature computational biology and integrate data science components across the majority of their educational offerings (101). This initiative aligns with Fiocruz’s Open Access to Knowledge policy, fostering collaboration and sharing among learners and partner institutions. Moreover, the Virtual Campus supports ongoing self-assessment and monitoring of its educational offerings, exemplified by its recent e-book publication on specialisation course evaluations and a continuous graduate survey, which are integrated into the institution’s academic management system. Additionally, Fiocruz has recently published a report on the Training Programme in Data Science and Health Information, highlighting its commitment to advancing data science capacity.

Fiocruz also supports a variety of health-related programmes that contribute to the advancement of the Brazilian Unified Health System (SUS) A noteworthy example is the Centre for Data and Knowledge Integration for Health, abbreviated to Cidacs, that operates under Fiocruz as a centre for interdisciplinary research and uses extensive databases (big data) to address complex health challenges. Cidacs is supported by international funding and overseen by an Executive Committee responsible for rigorous monitoring and evaluation of training programmes to ensure sustainability and impact. Cidacs’ key focus areas include bioinformatics, computing, epidemiology and statistics (102, 103). The main objectives of Cidacs are to: conduct innovative studies and research using interdisciplinary approaches; integrate large databases for insights into health issues; promote professional and scientific development with integrated training on big data and computing skills; facilitate knowledge integration at multiple levels; foster collaboration with national and international partners to stay abreast of technological advancements; and support evidence-based decision-making in public policies for improved health outcomes (102, 103). By harnessing knowledge and advanced computing resources, Cidacs contributes to a deeper understanding of health problems, driving positive changes in public health and the SUS system.

Fiocruz has a structured approach to health data science capacity building, leveraging open-source and free online courses through the Virtual Campus to promote resource sharing, and fostering innovation, knowledge exchange and collaboration via their dedicated centre at Cidacs. This structured framework enables sustainability, with Cidacs’ activities supported by rigorous monitoring and evaluation from its Executive Committee and sustained through diverse international funding.

#### 3.4.5 Open University of the Brazilian National Health System (UNA-SUS)

The UNA-SUS, initiated in 2010 by the Brazilian Ministry of Health, serves as an open university for the publicly-funded Brazilian Health System, SUS (104, 105). UNA-SUS strives to elevate the standard of training and continuing education for healthcare professionals by collaborating with institutions within the UNA-SUS Network. The programme adapts to the requirements of the populace, addressing diverse public health issues ranging from specialised topics, such as disease diagnosis and treatment, through to public health policies (104, 105). UNA-SUS also promotes the dissemination of information and communication tools, aiming to expand the scale and scope of educational efforts. Moreover, it contributes to reducing regional disparities within Brazil by providing training programmes and ongoing education opportunities across the country (104, 105).

The UNA-SUS programme’s goals are achieved through key centres, notably a consortium of higher education institutions. Currently, 35 institutions are involved, providing complimentary courses that have over 8 million enrolments for 480 offerings nationwide. Integral elements of the UNA-SUS system include the Health Educational Resources Collection (ARES) (106), a public digital repository, and the Arcoura platform. The former facilitates unrestricted internet access to technology, educational content and materials, thus constituting the largest digital health collection in Latin America; and the Arcoura platform functions as a national SUS database that accumulates all course-related data. Participants can navigate this platform based on their profession, interests, and geographical region, and it keeps records of educational certificates for SUS health professionals (107, 108).

Within the Brazilian Ministry of Health, there are other initiatives similar to UNA-SUS in operation. Notable examples include UniverSUS (109, 110) and AVA-SUS (111, 112). UniverSUS, financed by Ministry of Health, focuses on delivery of free remote courses in health information and informatics, and provides training and support for the advancement, assessment and administration of related distance learning initiatives. The programme is aimed at students, researchers and healthcare professionals involved in health informatics and information management (109, 110). Additionally, AVA-SUS provides another digital platform for distance education, aiming to enhance the skills of healthcare workers, professionals, students and educators, to strengthen the overall quality of healthcare in Brazil. UNA-SUS’s approach ensures a continuous national flow of knowledge production by sharing educational resources produced within the network. These materials, which include videos, texts, and audios, can be reused, remade or adapted, fostering a collaborative and resource-rich environment for healthcare education. This approach is further supported by the transparent sharing of metrics from the AVA-SUS platform itself, which helps in tracking progress and outcomes. Through interactive educational technologies and the integration of a virtual learning environment, AVA-SUS contributes to the qualification and recognition of an expanding workforce (111, 112).

The reliance on digital platforms necessitates robust internet infrastructure and accessibility, which can be uneven across different regions of Brazil. Moreover, ensuring that educational content remains updated and relevant requires ongoing collaboration and investment. However, the transparent metrics, continuous knowledge production, and collaborative framework significantly contribute to its sustainability and positive impact on Brazil’s healthcare system.

## 4 Discussion

This review of ongoing data science capacity building initiatives relevant to global health research in Africa, Asia and LAC has highlighted a notable growth in recent years towards such initiatives being developed and tailored for a diverse range of specific health research areas. These areas include infectious diseases, nutrition, maternal and child health, pandemic surveillance, genomics, non-communicable diseases, climate change and health, and the digitalisation of health systems. This approach ensures that initiatives align with and address regional and country health priorities, which is vital for effective implementation. The increase in data science capacity building initiatives has also been influenced by significant strategic drivers, such as the WHO’s strategy for digital health transformation and the contribution they can make to achieving the Sustainable Development Goals (SDGs). Furthermore, the COVID-19 pandemic underscored the critical importance of data science approaches in responding to global health crises.

Many of the current data science capacity-building initiatives identified operate through collaborative consortia and networking hubs that aim to facilitate effective and sustainable knowledge exchange. This multidisciplinary approach ensures that health data science training opportunities cover a wide range of skills, empowering researchers and health practitioners to tackle regional challenges, whilst contributing to a global community of practice. However, implementation and impact monitoring strategies vary across organisations, with each adopting its own framework and metrics, typically published in annual reports. For many initiatives, there is limited information available on how they evaluate and report on their impact. Furthermore, most organisations with published reports do not explicitly detail how their data science capacity building activities have enabled high-impact global health research. This clarity is crucial, particularly for funders so they can channel their investment into impactful data capacity building initiatives.

Several initiatives identified have adopted a multilayer approach to data science capacity building, emphasising the importance of engagement with a wide range of stakeholders, including government agencies, academic institutions and the private sector, as well as building robust data science infrastructure. Collaboration with government agencies facilitates policy integration and resource allocation, whilst partnerships with academic institutions foster knowledge exchange and research collaborations that utilise data science approaches and private sector involvement can leverage technological expertise and financial resources, enhancing programme scalability and innovation. Therefore, it is crucial to focus on data capacity building not just at the individual level, but also at the organisational and network levels, further strengthening the sustainability and impact of these initiatives.

In considering successful approaches, initiatives such as H3Africa, D-SI Africa, Fiocruz (Cidacs), and the Capacity Building Accelerator Network stand out for their exemplary role in creating robust ecosystems for data science capacity building in health research across a range of LMICs and regions. These initiatives adopt a multi-layered strategy that includes building data infrastructure alongside strengthening data science capacity. By engaging with a wide range of stakeholders, from health researchers to policymakers, they maximise both health and social impact whilst addressing country-specific needs. Furthermore, these initiatives secure dedicated funding through their highly collaborative networks and have also clear approach to evaluation, continuous improvement, governance frameworks and impact assessment further enhancing their effectiveness, scale and sustainability.

Despite their ambitious goals and practical benefits, data capacity building initiatives face challenges related to implementation, impact and sustainability. A primary issue is the variability in technological infrastructure and resource availability across the countries participating in multi-country consortia, which can impede the consistent application of training and best practices. In addition, the lack of robust impact assessment and tracking mechanisms make it difficult to measure tangible improvements in health information systems and public health outcomes. Sustainability is another critical challenge, as the long-term success of these initiatives depends on continuous funding, political commitment and ongoing capacity building. There is also a need for a stronger emphasis on building local capacity and ownership to ensure sustained improvements.

Whilst several organisations have impact evaluation mechanisms in place, greater coordination across initiatives and geographies is necessary. Adopting common health data science capacity building frameworks, such as the ESSENCE framework (112), or developing a new one could help funders channel investments more effectively, avoid duplication, align investments with other funders, and harmonise efforts based on regional needs and priorities. In addition, although this review focuses on data science capacity building initiatives in LMICs to empower local health researchers and practitioners, the significance of knowledge sharing and resource availability extends globally. Data science is a global community, and the shared learning experiences from these initiatives can also benefit high-income countries, highlighting a mutual interest in collaborative growth and development.

To our knowledge, this is the first review that attempts to identify and consolidate in one paper current global and regional health data science capacity building initiatives that are highly relevant for health researchers and practitioners in LMICs across Africa, Asia and LAC. It also identifies gaps within existing initiatives, as well as the critical success factors that ensure sustainability and impact, and makes recommendations to enhance the outcomes of health data science capacity building initiatives.

However, it is important to recognise the limitations of this review. Due to the limited number of publications available on the specific topic under consideration, we opted not to follow a formal systematic review methodology. Moreover, there is a notable absence of explicit information in the literature regarding how institutions and organisations integrate data science capacity building programmes into their health research initiatives, particularly in terms of plans available online, as well as the processes for scaling up and sustaining these initiatives. Furthermore, it should be noted that this is not normally a topic for publication, which explains the scarcity of coverage in the literature, whilst aligning with the objective of this paper to raise awareness. Additionally, the use of key words and terms only in the English language may have excluded data science capacity building initiatives reported in other languages, potentially limiting the scope of this review.

We also acknowledge that our review may have overlooked significant information, not solely due to language barriers, but also because some countries prioritise academic capacity building over organisational initiatives. Despite the growing traction of the latter approach, as evidenced by our review, it highlights the dynamic and diverse landscape of health data science. This aligns with our overarching aim to emphasise the necessity of integrating data science capacity building within health research, in response to the continually evolving nature of the data science field and ultimately, to accelerate research insights.

## 5 Conclusion and future directions

This review has identified ongoing data science capacity building initiatives relevant to health research within major organisations and institutions across LMICs. By examining the current landscape, it has sought to offer valuable insights into the data science and health research ecosystem globally, as well as within Africa, Asia and LAC, which may inform approaches, policies and investment decisions for data science capacity building programmes to accelerate the generation of valuable insights from global health research. Future initiatives should adopt structured approaches that prioritise the development of standardised frameworks with common implementation and monitoring mechanisms to guide data science capacity building efforts for global health research, as well as the use of robust impact measurements to guide investment in successful models of data science capacity building.

Furthermore, adopting a multi-layered approach to capacity building is vital for ensuring sustainability. This approach should integrate data science training with the development of robust data infrastructure, tailored to regional needs. Equally important is the engagement of diverse stakeholders, including health researchers, practitioners, policymakers and funders, to maximise the overall impact. In addition, making interdisciplinary connections between different sectors and disciplines will be crucial for promoting knowledge exchange and collaboration in both capacity building initiatives and data science-focused health research, as well as ensuring appropriate geographical coverage, representation and accessibility to help reach diverse research communities and address regional disparities in health data science capacity building.

## Footnotes

^1^ thegraphcourses.org
