# Supplementary material for: Current data science capacity building initiatives for health researchers in LMICs: global & regional efforts
Source: Front Public Health. 2024 Nov 27;12:1418382. doi: 10.3389/fpubh.2024.1418382 (PMC11631614; doi:10.3389/fpubh.2024.1418382)
Supplement: Supplementary file 1 [file Table_1.docx]

**Supplementary Table 1. Literature Review String Search Based on Database**

| **Database** | **String Search** |
| --- | --- |
| PubMed | (("capacity building" OR "capacity strengthening" OR "training") AND ("global health" OR "health data" OR "health data science" OR "health data research" OR "digital health" OR "healthcare" OR "disease surveillance" OR "data management" OR "genomics" OR "bioinformatics" OR "infectious disease" OR "non-communicable disease" OR "nutrition" OR "maternal health" OR "childhood health" OR "health systems") AND ("initiatives" OR "consortium" OR "network" OR "data science hub") AND ("LMICs" OR "low and middle income countries" OR "Africa" OR "Latin America and the Caribbean" OR "LAC" OR "South-East Asia" OR "South Asia" OR "low-resource settings")) AND ("2019/01/01"[Date - Publication] : "2024/01/1"[Date - Publication]). |
| Scopus | (TITLE-ABS-KEY("capacity building" OR "capacity strengthening" OR "training") AND TITLE-ABS-KEY("global health" OR "health data" OR "health data science" OR "health data research" OR "digital health" OR "healthcare" OR "disease surveillance" OR "data management" OR "genomics" OR "bioinformatics" OR "infectious disease" OR "non-communicable disease" OR "nutrition" OR "maternal health" OR "childhood health" OR "health systems") AND TITLE-ABS-KEY("initiatives" OR "consortium" OR "network" OR "data science hub") AND TITLE-ABS-KEY("LMICs" OR "low and middle income countries" OR "Africa" OR "Latin America and the Caribbean" OR "LAC" OR "South-East Asia" OR "South Asia" OR "low-resource settings")) AND PUBYEAR > 2019 AND PUBYEAR < 2023 |
