# Supplementary material for: Current data science capacity building initiatives for health researchers in LMICs: global & regional efforts
Source: Front Public Health. 2024 Nov 27;12:1418382. doi: 10.3389/fpubh.2024.1418382 (PMC11631614; doi:10.3389/fpubh.2024.1418382)
Supplement: Supplementary file 2 [file Table_2.docx]

**Supplementary Table 2. Current Health Data Science Capacity Building Initiatives: Global and Regional**

| **Name of Initiative** | **Organisation** | **Regional Focus** | **Main Focus** | **Data Science Skill Training Focus** |
| --- | --- | --- | --- | --- |
| Food Systems Transformation | The Global Partnership for Sustainable Development Data (GPSDD) | Global | Advancing data science in LMICs across multiple sectors including health. | Data access, variety of data methodologies and analysis |
| Division of Data, Analytics and Delivery of Impact (DDI) | WHO | Global | Promoting best data science practices for the delivery of reliable results utilised for driving evidence-based policy reforms and impact tracking. | Variety of Digital Health Topics |
| Disaster Risk Management Research Network (Health EDRM RN) | WHO | Global | Knowledge exchange of up-to-date evidence and comprehensive information on Health EDRM. | Health Data Management |
| The WHO hub for Pandemic and Epidemic Intelligence | WHO | Global | Mitigate the effects of pandemic and epidemic risks through a collaborative surveillance between nations and communities. | Data access, surveillance and analysis |
| Capacity Accelerator Network (CAN) | DATA.org | Global | Build data capacity accelerators in LMICs that will enable the training of the next generation of data practitioners by 2032, in the sphere of climate change and health. | Data analysis of epidemiological/surveillance data |
| Global Epidemic Response of the Future (Epiverse) | DATA.org | Global | Create a reliable data analysis ecosystem for monitoring infectious disease outbreaks. | Data analysis |
| Digital Health and Data | PATH | Global | Support LMICs in the development and advancement of the digital health transformation and data use. | All aspects of data use and access, including data collection, quality, management and analysis |
| The Global Research and Analyses for Public Health (GRAPH) Network | University of Geneva's Global Health Institute  &  WHO African Regional Office | Global | Develop open-source data science software, generate educational materials, and undertake research to advance global health initiatives. | Affordable live boot camps and open access courses, self-paced data analysis, Statistics, visualisation and data reporting using R with a focus on epidemiological/health datasets. |
| The Demographic and Health Surveys program (DHS) Learning Hub | DHS | Global | Collect and analyse data on population, health, and nutrition across LMICs. The DHS hub focused on data science capacity building, specifically on data analysis of survey data. | Open access courses, workshops and tutorials in survey data analysis. |
| Knowledge Sharing  Hubs | The Global Health Network  (TGHN) | Global | Facilitate research in LMICs through a network of knowledge sharing hubs, fostering knowledge exchange, methods and research findings across diseases, regions, and organisations. | Regional and online open access training, resources, and professional development opportunities to enhance skills and careers across disciplines. |
| European and Developing Countries Clinical Trials Partnership (EDCTP) Hub | The Global Health Network (TGHN) | Global | Provide resources and support on capacity development and awareness on data sharing, for clinical resource in LMICs. | Protocol Development Toolkit, Data sharing Toolkit and Data management portal. |
| The Global Health Data Science Hub (GHDSH) | The Global Health Network (TGHN) | Global | Provide open-access knowledge exchange platform for data scientists in health research. | Open-source training resources for health data research programming language (R). |
| The multi-country Human Heredity and Health in Africa (H3Africa) Consortium | H3Africa | Africa | Facilitate fundamental genomic research into African populations and developing infrastructure, resources, training, and ethical approaches to foster the growth of a sustainable research landscape in Africa. | Bioinformatics and biostatistics training |
| H3BioNet | H3Africa | Africa | Advance bioinformatic research capacity in Africa for the H3Africa Consortium, by providing support on training and data analysis, and access to bioinformatics infrastructure. | Bioinformatics and biostatistics training |
| Data Science for Health Discovery and Innovation in Africa (DS-I Africa) | DS-I-Africa | Africa | Leverage data science technologies to create innovative solutions for addressing the critical public health challenges facing the continent. | Research training programmes focused on research design, methods and analytic techniques and novel data science solutions. |
| Africa Centres for Disease Control and Prevention (Africa CDC) | Africa CDC | Africa | Support member states in advancing their public health initiatives and strengthen the capacity of African health institutions to effectively tackle disease threats. | Across the data science lifecycle |
| Institute of Pathogen Geonomics | Africa CDC | Africa | Strengthen global disease surveillance and public health collaborations by establishing integrated, cross-continental laboratory network and provide them with tools, personnel, and data infrastructure to optimize the use of crucial genomic sequencing technologies. | Bioinformatic approaches based on genomic sequencing approaches |
| West Africa International Centres of Excellence for Malaria Research (ICEMR) | ICEMR | Africa | Advance research efforts on malaria control, treatment, and prevention within the Sub-Saharan West African regions. | Data collection, management and analysis |
| World Health Organization: Regional Office for South-East Asia -Regional Strategic Roadmap on Health Security and Health System Resilience for Emergencies 2023-2027 | WHO | Asia | Strengthen national health security and enhance the resilience of health systems during emergencies, particularly regarding infectious hazards and enhance capacities at the regional level. | Robust systems and capacity strengthening for collecting and analysing big data, through the creation of predictive models |
| Tropical Health Network | Mahidol Oxford Tropical Medicine Research Unit (MORU | Asia | Accelerate public health and clinical research insights and developing cost-effective interventions that contribute towards the wellbeing of communities in low resource countries in Asia. | Geospatial data for disease surveillance, Mathematical and economic models, requiring advanced data science capabilities and contributions to curriculum development and instruction in MSc programmes through the Department of Mathematical and Economic Modelling (MAEMOD). |
| International Centre for Diarrhoeal Disease Research, Bangladesh (icddr,b)-  Strategic plan for 2023-2027 | Icddr,b | Asia | Expand the capacity to harness cutting-edge technological advancements in support of public health research | Big data and Machine Learning |
| The Asia eHealth Information Network (AeHIN) | AeHIN | Asia | Advance interoperability for improved healthcare | Resource sharing and knowledge exchange. |
| The Asia Pacific Malaria Elimination Network (APMEN) | APMEN | Asia | Eradicate malaria in the Asia Pacific region by 2030. | Data quality, integration, and technology |
| India Data Capacity Accelerator | Data.org | Asia | Equip data researchers and practitioners with the skills needed to address climate-related health challenges stemming from the ongoing climate crisis. | Multidisciplinary academic training to enhance data science capabilities |
| Integrated Disease Surveillance Project (IDSP) | National Health Mission for all States & Union Territories (UTs) in India | Asia | Strengthen a decentralised, laboratory-based IT model that enables the surveillance of epidemic-prone diseases through computational systems | Disease surveillance, data entry, outbreak reporting and data analysis |
| Pan American Health Organisation (PAHO)-  Strengthen Information Systems for Health 2019-2023 | PAHO | Latin America & Caribbean | Improve data capture and management capabilities in the health sector for improved decision-making, policy development, monitoring and evaluation | Digital literacy programmes, including digital health workshops and training on emerging digital technologies. |
| The Latin American and Caribbean Network for Strengthening Health Information Systems (RELACSIS) | PAHO | Latin America & Caribbean | Improve the quality of data, diagnostics and health policies in the region, covering all stages of data collection and analysis. | Best practices training on enhancing health information systems such as ICD-10 coding practices for medical information and extensive training package on ICD-11. |
| Health Informatics Association for Latin-America and Caribbean (IMIA-LAC) | International Medical Informatics Association (IMIA) | Latin America & Caribbean | Develop and strengthen health informatics in the LAC region. | Establishing academic programme in health informatics. |
| Oswaldo Cruz Foundation (Fiocruz)-Virtual Campus | Fiocruz | Latin America & Caribbean | Promote knowledge and learning in health education to strengthen the Brazilian public health system and improve the quality of life on the wider region. | Computational Biology course and include data science components across the majority of their educational offerings. |
| The Knowledge and Data integration Centre for Health (Cidacs) | Fiocruz | Latin America & Caribbean | Undertake innovative interdisciplinary studies, utilize large databases for health insights, and advance professional and scientific development through integrated training in big data and computing skills. | Bioinformatics, epidemiology, computer science and statistics training. |
| The Unified Health System-Brazil (UNA-SUS) | Brazilian Ministry of Health | Latin America & Caribbean | Elevate the standard of training and continuing education for healthcare professionals by collaborating with institutions within the UNA-SUS Network. | Unrestricted internet access to technology, a variety of educational content and materials, constituting the largest digital health collection in Latin America |
| UniverSUS | Brazilian Ministry of Health | Latin America & Caribbean | Offer complimentary remote courses in health information and informatics, including training and support for the development, evaluation, and management of distance learning initiatives. | Health Information management and Informatics |
| AVA-SUS | Brazilian Ministry of Health | Latin America & Caribbean | Enhance the skills of healthcare workers, professionals, students and educators, to strengthen the overall quality of healthcare in Brazil. | Educational material on a wide range of health disciplines including data science aspects |
